# Association of the Adipokines Chemerin, Apelin, Vaspin and Omentin and Their Functional Genetic Variants with Rheumatoid Arthritis

**DOI:** 10.3390/jpm11100976

**Published:** 2021-09-29

**Authors:** Alaa S. Wahba, Maha E. Ibrahim, Dina M. Abo-elmatty, Eman T. Mehanna

**Affiliations:** 1 Department of Biochemistry, Faculty of Pharmacy, Suez Canal University, Ismailia 41522, Egypt; dina_abouelmouti@pharm.suez.edu.eg (D.M.A.-e.); eman.taha@pharm.suez.edu.eg (E.T.M.); 2 Department of Physical Medicine, Rheumatology and Rehabilitation, Faculty of Medicine, Suez Canal University, Ismailia 41522, Egypt; maha_emad83@yahoo.com

**Keywords:** rheumatoid arthritis (RA), chemerin, vaspin, apelin, omentin

## Abstract

Adipokines were shown to exert crucial roles in rheumatic diseases. This study aimed to assess the role of chemerin, apelin, vaspin, and omentin adipokines and their genetic variants rs17173608, rs2235306, rs2236242, and rs2274907, respectively, in rheumatoid arthritis (RA) pathogenesis in Egyptian patients. A total of 150 RA patients and 150 healthy individuals were recruited. Blood samples were collected and used for genotyping. Serum was separated and used for expression analysis by quantitative PCR, and various biochemical markers determination by ELISA. Serum protein levels of chemerin and vaspin, as well as their gene expression levels were higher, while those of apelin and omentin were lower in RA patients and were associated with most of RA clinical and laboratory characteristics. G allele of chemerin rs17173608, T allele of vaspin rs2236242, and T allele of omentin rs2274907 were more frequent in RA patients. Serum levels and gene expression levels of chemerin in GG genotype carriers and vaspin in TT genotype group were significantly higher, while those of omentin in TT genotype carriers were significantly lower than RA patients with other genotypes. There was no association between apelin rs2235306 and RA. Chemerin rs17173608, vaspin rs2236242, and omentin rs2274907 polymorphisms were associated with increased susceptibility to RA.

## 1. Introduction

Rheumatoid arthritis (RA) is an inflammatory autoimmune disorder with 1% prevalence worldwide. RA is characterized by severe joint injury and cartilage and bone destruction, eventually leading to impaired physical function and quality of life. It affects genetically susceptible individuals with the involvement of environmental factors and epigenetic mechanisms [1]. RA is characterized by the presence of rheumatoid factor (RF) and anticitrullinated protein antibody (ACPA) in the majority of patients [2].

The complex interplay between these triggering factors elicits protein citrullination, thereby creating neoantigens which trigger immune response in genetically susceptible individuals leading to production of anticitrullinated peptide antibodies (ACPAs). This stage, loss of tolerance, is usually followed by clinical synovitis [3]. Clinical synovitis is characterized by augmented vascular permeability and infiltration of inflammatory cells into the synovium. As a result, fibroblast-like synoviocytes (FLSs) acquire an altered phenotype secreting a diverse array of proinflammatory cytokines, chemokines, and matrix metalloproteinases (MMPs), leading to chronic inflammation, synovial hyperplasia, and progressive joint and cartilage destruction and resultant disability [4].

Interestingly, adipocytokines, including adipokines, cytokines, chemokines, and complementary factors are involved in regulation of glucose and lipid metabolism, blood pressure, and modulation of immune response and inflammation, and thus, are implicated in various physiological and pathological processes [5,6]. Adipokines are not exclusively produced by the white adipose tissue, but also are synthesized by immune cells, chondrocytes and synoviocytes with crucial roles in rheumatic diseases both locally and systemically [7]. Previous studies demonstrated the role of the adipokines in the pathogenesis of RA, exerting anti- as well as proinflammatory functions and modulating autoimmune responses. Accumulating evidence has revealed the possibility to use adipokines’ circulating levels as potential biomarkers of disease activity and therapeutic response. In view of these concerns, better understanding of the mechanisms involved in their dysregulation is crucial for the development of successful treatment approaches to achieve remission or low disease activity in RA [8].

As a result, any genetic variation in adipokine genes that affect their levels may contribute to various pathophysiological conditions. Association between adipokine single-nucleotide polymorphisms (SNPs) and their respective adipokine levels [9], as well as the risk of obesity, insulin resistance, dysregulation of lipid metabolism, type 2 diabetes mellitus (T2D), metabolic syndrome (MetS), and inflammatory diseases was recognized [10,11,12]. This study focused on the of the adipokines chemerin, apelin, vaspin, and omentin in RA and the association of their functional genetic variants with increased susceptibility to the disease. 

Chemerin is an adipokine encoded by retinoic acid receptor responder protein 2 (RARRES2) gene, also described as tazarotene-induced gene 2 (TIG2) and located at 7q36.1 in humans [13]. Chemerin is secreted as an inactive proprotein that is subsequently converted to the active protein by posttranslational C-terminal cleavage mediated by several proteases giving rise to different chemerin functions [14]. Chemerin was implicated in immune and metabolic homeostasis [15]. In addition, it was demonstrated to exert direct proinflammatory effects on the RA-FLS and enhance immune cells infiltration, suggesting its implication in RA pathogenesis [15,16]. The intronic rs17173608 SNP of the RARRES2 gene was related to an increased risk of obesity and MetS [17,18,19]. 

Apelin, another adipokine, is coded by gene localized on chromosome Xq25–q26.1 which comprises 3 exons, with its coding region spanning exons 1 and 2 [20]. It was suggested that apelin enhances insulin sensitivity, delays development of obesity associated metabolic disorders, and acts as nitric oxide (NO)-dependent vasodilator [21,22,23]. Previous studies demonstrated that decreased apelin levels in RA patients may be associated with increased cardiovascular risk [24,25]. However, the exact role of apelin in modulating inflammatory responses remains undefined. A previous study demonstrated that apelin, through c-Jun N-terminal kinases (JNK) and nuclear factor kappa B (NF-κB) activation, induces inflammation [26]. Another study reported that apelin attenuates inflammation and improves diabetic nephropathy through decreased monocytes infiltration, and NF-κB activation [27]. The apelin rs2235306, an intronic SNP, was shown to be associated with insulin secretion, and fasting glucose level [28,29].

Vaspin, visceral adipose tissue-derived serine protease inhibitor, is coded by SERPNA12 gene located on 14q 32.13 [30]. Vaspin exerts insulin-sensitizing effects [31], as well as anti-inflammatory and antiapoptotic effects [32]. Increased vaspin levels were reported in diabetes and obesity suggesting its role as a compensatory mediator against obesity induced inflammation [33]. In addition, previous findings reported elevated vaspin levels in synovial fluid of RA patients indicating possible involvement during arthritis development [34,35]. It was reported that the intronic SNP rs2236242 was associated with increased risk to MetS [18,19], and T2D [36].

Omentin-1, also known as intelectin-1, is another adipokine coded by gene located at chromosome 1q 21–23 [37,38]. It was reported to exert insulin sensitizing, potential anti-inflammatory, antiatherosclerotic, and cardiovascular protective effects [39]. Several lines of evidence showed that reduced omentin-1 levels are associated with inflammatory and autoimmune diseases including diabetes, atherosclerosis, and RA [40]. The rs2274907 (A326T) SNP lies within exon-4 of the omentin 1 gene, where at position 109, aspartate is exchanged with valine (Val109Asp or V109D). An association between omentin rs2274907 and T2D, obesity, and cardiometabolic diseases was previously reported [41].

The aim of this study was to investigate the frequency of the molecular variants, the gene expression, and the circulating levels of chemerin, apelin, vaspin and omentin 1 in RA Egyptian patients compared to healthy individuals and to assess their association with RA.

## 2. Materials and Methods

### 2.1. Study Participants

The study involved 150 (60 males, 90 females) unrelated RA patients (mean age: 44.29 ± 9.4 years) and 150 (75 males, 75 females) apparently healthy controls (mean age: 42.07 ± 11.3 years) with no history of RA, or any other chronic diseases. The two groups of patients and control were gender matched (*p* = 0.08) and age matched (*p* = 0.07). The sample size required to detect the association between SNPs and RA was calculated using formula described by Charan and Biswas [42] with a power of 80% and level of significance of *p* < 0.05. All participants were of the same ethnic group minimizing the potential for population stratification. RA patients were enrolled during the period between September 2019 and September 2020 at the outpatient Rheumatology and Orthopedic Clinics, Suez Canal University Hospitals, Ismailia, Egypt. RA was defined according to the 2010 American College of Rheumatology (ACR)/European League Against Rheumatism (EULAR) classification criteria of RA [43]. The exclusion criteria for the patients were presence of pregnancy, current infection, endocrine diseases, cardiac diseases, diabetes mellitus, current renal or liver disease, and any other autoimmune diseases.

The patients underwent a full history and clinical examination. Anthropometric assessment included weight and height measurements from which the body mass index (BMI) was calculated. There was no significant difference between the mean BMI for RA patients (26 ± 2.7) (kg/m^2^) and that of control subjects (25.44 ± 2.4) (kg/m^2^) (*p* = 0.06). Disease Activity Score of 28 joints (DAS-28) was calculated based on the swollen joint count, tender joint count, C-reactive protein (CRP) and visual analog scale (VAS) [44]. All RA patients were treated with Methotrexate (doses ranging from 12.5 to 20 mg/week) and/or Leflunomide (20 mg daily), synthetic disease modifying antirheumatic drugs (DMARDs), in addition to glucocorticoid therapy with the dose varied according to disease activity (median (IQR) = 5 (2.5–10) mg daily). None of the patients included in the study were on biological or targeted synthetic (ts) DMARDs. Table 1 presents the characteristics of the study participants.

### 2.2. Ethical Approval

The study protocol was approved by the Ethics Committee of Faculty of Pharmacy, Suez Canal University (201909RH2) and was performed in accordance with the principles of the Declaration of Helsinki (2000 revision). All participants signed written informed consent.

### 2.3. Biochemical and Immunochemical Analysis

For all participants, overnight fasting blood samples (6 mL) were drawn and divided into two portions: one portion (2 mL) was collected in an EDTA tube for DNA extraction and ESR testing using Westergren’s method. Serum was separated from the remaining portion, collected in a plain tube, and used for RNA extraction and assessment of the following parameters: serum CRP and IgM-rheumatoid factor (IgM-RF) using the immunonephelometry method, and anticyclic citrullinated peptide (anti-CCP) antibody using enzyme linked immune sorbent assay (ELISA) (anti-CCP high-sensitive ORG 601). Control subjects with abnormal levels of any of these parameters were excluded from the study. Cutoff value for RF and anti-CCP antibody was 20 IU/mL in accordance with producer’s instructions. Circulating chemerin, apelin, vaspin and omentin 1 levels were assessed using (RayBio^®^ Human Chemerin ELISA Kit, Cat No. ELH-Chemerin, CUSABIO Human Apelin ELISA Kit, Cat No. CSB-E14334h, RayBio^®^ Human/Mouse/Rat Vaspin ELISA Kit, Cat No. EIA-VAP, EIAM-VAP, EIAR-VAP, RayBio^®^ Human/Mouse/Rat Omentin ELISA Kit, Cat No. EIA-OME, EIAM-OME, EIAR-OME, respectively). Serum NF-κB, hypoxia-Inducible Factor (HIF-1α), MMP-3, vascular endothelial growth factor (VEGF) and Bcl-2–associated X (BAX) levels were detected using the human specific ELISA kits (BioSource International, CA, Cat No. MBS260718, Cat No. MBS261767, Cat No. MBS2500861, Cat No. MBS355343, Cat No. MBS701787, respectively). ELISA kits were used according to manufacturer’s instructions.

### 2.4. Genotyping

Whole blood was used for genomic DNA extraction using a Wizard Genomic DNA Purification kit (Promega Corporation, Madison, WI, USA) according to the manufacturer’s instructions. DNA concentration and purity were evaluated using NanoDrop ND-1000 (NanoDrop Tech., Inc., Wilmington, DE, USA). Chemerin rs17173608, apelin rs2235306 and vaspin rs2236242 SNPs were genotyped by the tetra-primer amplification refractory mutation system-polymerase chain (T-ARMS PCR) reaction, while omentin 1 rs2274907 polymorphism was detected by polymerase chain reaction- restriction fragment length polymorphism (PCR-RFLP). The primers, annealing temperatures, and band lengths are listed in Table 2. The amplification reactions were prepared by adding 1 μL of each primer (10 ρmol/μL), 1 μL of DNA template (25 ng/μL), 12.5 μL of GoTaq^®^ Green Master Mix (2x) (Promega, Madison, WI, USA) and made up to 25 μL final volume with DNAse-free water. Thermal cycling conditions were set as following: 5 min at 95 °C for initial denaturation, followed by 30 cycles of amplification: 1 min at 95 °C, annealing for 1 min, and 1 min at 72 °C, then a final extension for 10 min at 72 °C. Thermal cycling was performed in an Eppendorf Mastercycler^®^ machine (Eppendorf, Hamburg, Germany). After DNA amplification, PCR products were visualized by horizontal gel electrophoresis on a 2.0% agarose gel. In case of omentin 1 rs2274907; the 471 bp amplified band was digested by AccI restriction enzyme, where 10 μL of the PCR product was incubated with 5 units (0.5 μL) of the enzyme at 37 °C for 60 min followed by visualization of the resulting bands on 2.0% agarose gel by using an ultraviolet (UV) trans illuminator. The 471 bp was cut into 274 and 197 bp bands in the presence of the T allele but remained intact in the presence of the A allele. To assure the accuracy of genotyping of all the investigated SNPs, 20% of the samples were selected randomly and repeated, where the results were identical to the first call by 100%.

### 2.5. Adipokines Expression Analysis

Total RNA was isolated from serum samples using Qiagen miRNeasy Mini kit (Qiagen, Hilden, Germany) following manufacturer’s instructions. RNA was quantified using a NanoDrop ND-1000 spectrophotometer (NanoDrop Tech., Wilmington, DE, USA), then stored at −80 °C until use. The adipokines expression was quantified by real-time PCR which was performed in StepOnePlus™ thermal cycling instrument (Applied Biosystems, CA, USA) using the GoTaq^®^ 1-Step RT-qPCR kit (Promega, Madison, WI, USA), including negative control in all runs. Beta-actin (ß-actin) was used as housekeeping gene. The sequences of primers were the outer primers used for T-ARMS PCR for chemerin, apelin and vaspin and the same primers used for PCR for omentin 1, and Sense, CACCCAGCACAATGAAGATC; Antisense, GTCATAGTCCGCCTAGAAGC for ß-actin. Each reaction consisted of RNA template (4 μL), forward and reverse primers (1 μL each, 5 ρmol/μL), GoScript™ RT mix (0.4 μL), GoTaq^®^ qPCR master mix (10 μL), supplemental CXR reference dye (0.31 μL), and nuclease-free water (3.29 μL) to reach 20 μL final volume. The program was initiated with reverse transcription step at 37 °C for 15 min, followed by inactivation of reverse transcriptase at 95 °C for 10 min, and continued with 40 cycles of amplification: 10 s at 95 °C for denaturation, 30 s for annealing, and 30 s at 72 °C for extension. The annealing temperatures presented in Table 2 were also implied for the amplification reactions of chemerin, apelin, vaspin, and omentin 1, and 54 °C was assigned as an annealing temperature used for amplification of ß-actin. Relative gene expression levels were calculated using the 2-ΔΔCt method. To test qPCR reproducibility, 20% of the samples were randomly chosen and reassessed in separate runs.

### 2.6. Statistical Analysis

Statistical analysis was carried out using Graphpad prism software v.5 (GraphPad Software Inc., La Jolla, CA, USA) and SPSS, version 21.0. Normality of the continuous data was tested by Shapiro–Wilk test. Quantitative normally distributed variables were represented as mean ± standard deviation (SD), while non-normally distributed variables (non-parametric data) were represented as median (1st–3rd quartile). For normally distributed data, student’s t-test and one-way ANOVA with Tukey’s posthoc for multiple comparisons were used to compare between two and three groups, respectively. For nonparametric data, comparisons were done by Mann–Whitney U-test for the two groups and Kruskal–Wallis test for the three groups. Dunn’s test was used for pair-wise comparisons. Categorical variables, presented as frequencies, were analyzed using the chi-square (χ2). Odds ratios (OR) with a 95% confidence interval (CI) were calculated. Also, the Chi-square test was used to determine whether the genotype distribution was compatible with Hardy-Weinberg equilibrium. One-way multivariate analysis of variance (MANOVA) was performed to evaluate the effect of RA on the serum and expression levels of the investigated adipokines and other determined biomarkers. Pearson and Spearman correlations between different variables were performed. A *p* value less than 0.05 was considered statistically significant.

## 3. Results

### 3.1. Serum Disease Biomarkers and Adipokines Protein and Expression Levels in the Enrolled Subjects

As shown in Table 3, serum levels of NF-κB, HIF-1α, MMP-3 and VEGF were significantly higher by 4.34-, 6.37-, 6.61-, and 2.95-fold in RA patients than healthy individuals, respectively (*p* < 0.001), whereas serum BAX levels were significantly reduced by 0.49-fold in RA patients compared to that of healthy individuals (*p* < 0.001). In addition, serum protein levels of chemerin and vaspin, determined by ELISA, were found to be significantly higher by 2.49- and 1.86-fold, respectively (*p* < 0.001), while serum apelin and omentin levels were found to be lower by 0.58- and 0.25-fold, respectively (*p* < 0.001) among RA patients relative to controls.

Median CT values of the gene expression of chemerin, apelin, vaspin, and omentin, determined in serum by qPCR, varied significantly between control and patients (*p* < 0.01). Up-regulation of chemerin and vaspin gene expression was evident in 59.3% and 94.7% of patient samples, respectively. The median gene expression levels of chemerin and vaspin in patients varied significantly by 2.3- and 6.80-fold, respectively compared to that of control. On the other hand, there was a downregulation of apelin and omentin gene expression in 93.3% of patient samples. The median gene expression levels of apelin and omentin in patients varied significantly by 0.35- and 0.21-fold, respectively compared to control.

Finally, multivariate analysis was performed to assess the simultaneous effect of RA on serum levels of NF-κB, HIF-1α, MMP-3, VEGF, BAX, chemerin, apelin, vaspin, and omentin, as well as the serum gene expression levels of the investigated four adipokines (Table 4). Multivariate analysis of variance (MANOVA) indicated that RA had a significant impact on the serum levels of NF-κB, HIF-1α, MMP-3, VEGF, and BAX [partial eta squared = 0.888, 0.879, 0.888, 0.936 and 0.711, respectively] (*p* = 0.001). The incidence of RA had also a significant impact on the serum and expression levels of chemerin (partial eta squared = 0.935 and 0.103, respectively), apelin (partial eta squared = 0.966 and 0.414, respectively), vaspin (partial eta squared = 0.968 and 0.191, respectively), and omentin (partial eta squared = 0.794 and 0.685, respectively) (*p* = 0.001). Notably, partial eta squared values indicate the large effect size of RA on the serum protein levels of all the investigated biomarkers (Table 4).

### 3.2. Association of Disease Biomarkers and Adipokines Protein and Expression Levels with Clinical and Laboratory Characteristics of RA Patients

BMI, DAS-28, ESR, CRP, RF, and anti-CCP antibodies were significantly positively correlated with serum levels of NF-κB, HIF-1α, MMP-3, and VEGF and significantly negatively correlated with serum BAX levels (*p* < 0.001). Moreover, there was a significant positive correlation of chemerin and vaspin serum protein and genetic expression levels with the clinical and laboratory characteristics of RA patients including BMI, DAS-28, ESR, CRP, RF, and anti-CCP antibodies (*p* < 0.05), except BAX, where a significant negative correlation was found (*p* < 0.001) (Table 5). On the other hand, apelin and omentin serum protein and gene expression levels were significantly negatively correlated with BMI, DAS-28, ESR, CRP, RF, and anti-CCP antibodies (*p* ≤ 0.01) with the exception of lack of significant correlation of apelin gene expression levels with BMI, RF and anti-CCP antibodies (*p* = 0.155, *p* = 0.07 and *p* = 0.051, respectively) and presence of a significant positive correlation with BAX levels (*p* < 0.001). In addition, adipokine gene expression levels were significantly positively correlated with their respective serum protein levels (*p* < 0.05). No significant correlation between any of the investigated biomarkers and age or sex of RA patients was observed (Table 5).

### 3.3. Molecular Analysis of Adipokines Genetic Variants in the Enrolled Subjects

The analysis included all participants. The distribution of the adipokines variants genotypes among patients and controls did not deviate from Hardy-Weinberg predictions (*p* > 0.05). SNP analysis of chemerin rs17173608, apelin rs2235306, vaspin rs2236242, and omentin rs2274907 showed that the incidence of chemerin minor G allele (*p* = 0.001; OR = 2.6), vaspin major T allele (*p* = 0.001; OR = 2.01) and omentin minor T allele (*p* = 0.016; OR = 1.52) was significantly higher in RA patients than in control group. Regarding apelin rs2235306, the distribution of major and minor alleles didn’t differ significantly between RA patients and control subjects (*p* = 0.325; OR = 1.18) (Table 6).

### 3.4. Adipokines Genetic Variants Association with their Corresponding Adipokine Protein and Expression Levels and Clinical and Laboratory Characteristics in RA Patients

The high-risk chemerin TG/GG genotypes’ groups were associated with a significant elevation in DAS-28, ESR, CRP, RF, anti-CCP antibodies, NF-κB, HIF-1α, MMP-3, VEGF and serum levels of chemerin (*p* < 0.05) and significant reduction in serum BAX levels (*p* < 0.001) in comparison with TT genotype carriers. On studying vaspin rs2236242 polymorphism, the high-risk TT/TA genotype carriers were found to have higher levels of DAS-28, ESR, NF-κB, and vaspin protein and lower serum BAX levels than AA genotype group at *p* < 0.05, while anti-CCP antibodies, MMP-3 and VEGF were higher in TT genotype only (*p* < 0.05). There was no significant difference in CRP, RF and HIF-1α levels among different genotypes (*p* = 0.05, *p* = 0.95 and *p* = 0.08, respectively). Moreover, DAS-28, ESR, CRP, anti-CCP antibodies, NF-κB, HIF-1α, MMP-3, VEGF were higher (*p* < 0.001), whereas serum BAX and omentin levels were lower (*p* < 0.01) in the high-risk omentin AT/TT genotype carriers as compared to the low-risk AA genotype group. RF was found to be significantly elevated only in omentin TT genotype carriers (*p* < 0.001). Also, there was a significant increase in relative expression of chemerin in GG genotype carriers and vaspin in TT genotype group, and on the contrary, significant decrease in omentin relative expression in TT genotype carriers when compared with that of RA patients with other genotypes (*p* = 0.02, *p* = 0.04 and *p* = 0.006, respectively). Regarding apelin rs2235306, no significant differences were observed in RA clinical and laboratory characteristics among different genotypes (Table 7).

## 4. Discussion

Chronic synovial inflammation is considered to be the main pathological feature that eventually leads to progressive joint destruction and comorbidities in RA. Initiation of inflammation is governed by immune cells infiltration into the synovium and activation of NF-κβ signaling in these cell types leading to enhanced transcription of target genes involved in immune and inflammatory responses [45,46]. Several lines of evidence indicates that NF-κβ deregulation plays a role in the pathogenesis of RA and other inflammatory diseases [47,48]. In agreement with these findings, this study reported significantly increased serum NF-κβ levels in RA patients compared to healthy subjects.

Fibroblast-like synoviocytes in the synovial lining exert a crucial role in RA associated synovial inflammation and joint destruction by secreting cytokines such as (TNF)-α, ILs, and MMPs, which mediate degradation of basement membrane and extracellular matrix protein, triggering tissue damage [49]. Thus, RA-associated cartilage and bone erosion is governed by MMPs upregulated production [50]. In line with these findings, current results revealed a significant rise in serum MMP-3 levels in RA patients.

Moreover, RA-FLSs acquire an altered phenotype showing an aggressive behavior characterized by enhanced proliferation and migration and delayed apoptosis leading to synovial hyperplasia, pannus formation, and chronic hypoxia [51]. Hypoxia enhances HIF1-α stabilization, translocation into the nucleus, and dimerization with its partner HIF1-β, thus activating the hypoxia signaling pathway which regulate cellular response to hypoxia through induction of HIF target genes transcription [52]. Consistent with these findings, this study showed significantly elevated HIF-1α serum levels in RA patients compared to healthy individuals.

Vascular endothelial growth factor is one of the HIF-1α target genes, which is upregulated to enhance oxygen supply to the hypoxic region [53]. A previous study demonstrated that oedema and joint swelling in RA were attributable to VEGF induced angiogenesis and increased vascular permeability [54]. In harmony with previous studies, there was a significant rise in VEGF serum levels in RA patients compared to that of control group in our study.

It was demonstrated that reduced blood and oxygen supply to the cells induces apoptosis due cellular energy deficiency, however persistent exposure to hypoxic conditions drives the cells to adapt to these conditions and to promote anti-apoptotic pathways through enhanced anti-apoptotic proteins expression including increase in Bcl-2 and Mcl-1 and attenuated pro-apoptotic proteins expression including BAX and Bid in a HIF-1 independent manner [55]. A growing body of evidence indicates that RA-FLSs are resistant to apoptosis and exhibit altered mitochondrial pathways of apoptosis, regulated by Bcl-2 family of proteins [4]. Previous studies demonstrated elevated expression of anti-apoptotic proteins Bcl-2 and Mcl-1 [56], and on the other hand, decreased proapoptotic proteins levels such as BAX in RA-FLS [57]. In accordance with these studies, our study exhibited significantly reduced BAX serum levels in RA patients compared to that of the control group.

Several studies emphasized the important role of adipokines, mediators secreted by adipose tissue, in RA [2]. Current results revealed that serum chemerin protein and expression levels were significantly higher in RA patients compared to that of healthy individuals, and these levels were positively associated with disease activity parameters and with serum NF-κB, HIF-1α, MMP-3 and VEGF, whereas they were negatively associated with serum BAX levels in RA patients, in addition, 59.3% of RA patients exhibited up-regulation of chemerin, suggesting a potential implication of this adipokine in RA.

A previous study reported that high chemerin levels in RA synovium are attributable to TNF-α and IFN-γ mediated upregulated production by FLSs, together with enhanced conversion of inactive prochemerin to active chemerin by extracellular protease produced by infiltrated neutrophils and mast cells in RA synovium [58]. Chemerin regulates inflammatory responses through activation of MAPK and PI3K/Akt pathways leading to enhanced production of inflammatory mediators by RA FLSs [58] and NF-κB activation [59], in addition to its chemoattractant properties enhancing immune cells infiltration into the RA synovium [16].

Moreover, previous studies demonstrated that hypoxia enhances chemerin expression, and this enhanced expression induces angiogenesis and new blood vessels formation in an attempt to counteract the effects of hypoxia [60,61]. Also, chemerin was reported to induce expression of degradative mediators as MMP-3 in RA FLSs [58] and prevent apoptosis via activating chemR23/ calmodulin-dependent protein kinase kinase 2 (CAMKK2)/ adenosine monophosphate-activated protein kinase (AMPK) signaling pathway. Activation of AMPK, homeostatic regulator of cellular energy levels, enhances glycolysis by phosphorylation and activation of 6-phosphofructo-2-kinase/fructose-2,6-bisphosphatases-3 (PFKFB3), ultimately protecting from death [62]. Chemerin was demonstrated to upregulate gene encoding PFKFB3 protein [63]. PFKFB3, also regulated by HIF-1α, was reported to be upregulated in multiple cancer types, where it was required for their survival and growth [64]. In addition, chemerin was demonstrated to cause resistance to programmed cell death by the attenuated expression of the proapoptotic proteins, cleaved caspase 3 and BAX [65].

Several studies provided evidence that polymorphisms in adipokines genes were associated with obesity, T2D and MetS risk [19,41,66]. We supposed that studied adipokines genes polymorphisms play a crucial role in RA pathogenesis. Current data revealed that there was a significant difference in genotype distributions and allele frequencies of chemerin rs17173608 between RA patients and control subjects, where chemerin minor G allele was more recurrent in RA patients than in control group. Also, relative expression of chemerin in GG genotype carriers was significantly higher when compared with that of RA patients with other genotypes, suggesting a possible functional role. Similarly, previous studies reported an association of chemerin SNPs with circulating chemerin levels and suggested ethnic genetic heterogeneity with distinct data for each ethnic population [67].

Regarding apelin, this study showed that its mRNA and protein levels are reduced in RA patients and inversely correlate with RA severity indices except that there was no significant correlation between apelin expression levels and RF and anti-CCP antibodies. Reduced apelin expression may be attributable to IL-6 mediated downregulation of bone morphogenetic protein receptor 2 (BMPR2) expression [68], leading to disruption of PPARγ/β-catenin complex, consequently impairing transcription of target gene APLN, that encodes apelin [69].

Moreover, apelin was found to have a potent anti-inflammatory activity, evidenced by attenuated inflammatory mediator production through preventing NF-κB binding to their promoter region; in addition, loss of apelin delayed IκB proteins replenishment, enhancing NF-κB activation [70]. Consistent with these findings, current results showed negative association between apelin and NF-κB levels. Also, this study showed inverse correlation between apelin and MMP3 levels in RA patients suggesting that loss of apelin promotes MMP-3 expression, which may be mediated through NF-κB induced transcription.

Interestingly, the apelin promoter region contains consensus sequences for HIFs leading to dramatic elevation in apelin expression during hypoxia as a compensatory and adaptive mechanism due to its implication in angiogenesis independent of the VEGF signaling pathway [71]. However, the response of the apelin pathway to hypoxia was reported to be biphasic with an initial upregulation followed by a normalization or downregulation [72], also we suggest that hypoxia induced enhanced apelin production is overwhelmed by BMP mediated inhibited expression. This study revealed an inverse association between apelin and both HIF-1α and VEGF levels in RA patients.

Apelin/APJ system was found to be implicated in apoptosis regulation and development of various diseases showing either proapoptotic or antiapoptotic activity depending on the onset and development of the disease [73]. A previous study indicated that apelin delayed the onset of apoptosis for a few hours in human enterocyte model (Caco-2), followed by enhanced apoptosis. The antiapoptotic effect of apelin was demonstrated to be as a result of enhanced anti-apoptotic Bcl-2 family proteins synthesis and interaction with phosphatidylinositol 3-kinase protein, whereas its proapoptotic activity may be attributable to suppressed expression of 8-oxoguanine DNA glycosylase1/2 (OGG1/2), DNA oxidative damage repair enzyme, leading to impaired oxidative DNA repair and induction of apoptosis through the mitochondrial apoptotic pathway [74,75]. A previous study reported elevated plasma levels of 8-hydroxy-2′-deoxyguanosine, the cleavage product of OGG enzyme, in RA patients indicating its higher activity to overcome the localized oxidative stress in the RA synovium [76]. These findings suggest that loss of apelin function gives rise to resistance to apoptosis. Similarly, current results revealed negative correlation between apelin and BAX levels.

However, current results did not find any significant relationship between apelin gene polymorphism rs2235306 and RA and no significant differences were observed in RA clinical and laboratory characteristics among different genotypes. In the same line, Mehanna et al. [20] found no association of apelin rs2235306 with metabolic syndrome or any of its component traits in Egyptian women in a previous study. Lack of association could be attributed to the variation of ethnicities, as this polymorphism was reported to be associated with the clinical features of diabetes mellitus in previous studies conducted on Iranian and Chinses populations [28,29].

Concerning the adipokine vaspin, this study’s data showed that serum vaspin protein and mRNA levels were significantly higher in RA patients compared to that of the control group, and this upregulation was evident in 94.7% of patient samples. In addition, vaspin levels were positively associated with disease activity indices and with serum NF-κB, HIF-1α, MMP-3, and VEGF, and on the contrary, they were negatively associated with serum BAX levels in RA patients.

Previous studies suggested that different chronic inflammatory diseases have different impact on vaspin levels [76]. Vaspin upregulation was found in obesity and T2D which was suggested to act as a compensatory mechanism and to exert anti-inflammatory action through binding to GRP78/MTJ-1, a cell-surface receptor complex, after being translocated to the plasma membrane in response to ER stress and protect against ER stress-induced metabolic disorders [77]. However, other studies documented that vaspin can also exert a proinflammatory role [78]. RA FLSs exhibit proinflammatory cytokines induced GRP78 expression that was reported to be crucial for RA FLSs survival and proliferation and enhanced angiogenesis. Binding of vaspin as a soluble ligand for plasma membrane GRP78 coreceptor activates the AKT/PI3K prosurvival pathway and prevents FLSs apoptosis subsequently leading to synovial hyperplasia, which further facilitates of cytokines secretion establishing a vicious cycle [79].

It was demonstrated that vaspin resulted in an enhanced antiapoptotic Bcl-2 expression and mitigated proapoptotic Bax expression through MAPK/ERK signaling pathway activation [80]. Thus, downstream signaling of vaspin GRP78/MTJ-1 pathways may induce NF-κβ activity and production of proinflammatory cytokines [81]. Similarly, current results suggest that vaspin contributes to the pathogenesis of RA.

In addition, this study showed significantly higher frequencies of rs2236242 (TT) genotype and major T allele in RA subjects group compared to control group. Moreover, the carriers of vaspin rs2236242 polymorphism TT genotype had significantly higher vaspin relative expression in comparison to that of RA subjects carrying other genotypes. In line with this finding, previous study reported that vaspin rs2236242 A allele is associated with lower vaspin levels impairing inflammatory/anti-inflammatory balance [82].

Another studied adipokine, omentin, was reported to be present in reduced levels in RA patients’ synovial fluids [34]. Similarly, this study data showed downregulation of omentin-1 expression in 93.3% of RA patient samples and significant decrease of serum omentin-1 protein levels in RA patients compared to healthy individuals. Also, it was negatively correlated with the measured RA clinical and laboratory indices except with BAX, there was positive correlation. Consistent with our findings, a previous study demonstrated that omentin has an anti-inflammatory role through inhibiting tumor necrosis factor α-induced IkBa degradation and consequently NF-κβ activity [83]. In addition, omentin-1 suppresses the JAK-2/STAT3 pathway activation, contributing to its anti-inflammatory role and ameliorating MMP expression [84]. Interestingly, omentin-1 concentration was found to be decreased in response to hypoxia [85], in line with current results which showed a negative correlation between omentin and HIF-1α levels, indicating that hypoxia could be the early trigger for altering omentin secretion. Decreased omentin-1 levels can lead to enhanced VEGF expression, and consequently cell migration and angiogenesis [86], in addition to increased resistance to apoptosis through decreased bax/bcl-2 protein ratio [87]. Furthermore, current results showed that omentin rs2274907 achieved significant difference in genotype distributions and allele frequencies between RA patients and controls. Results indicated that the omentin rs2274907 AT genotype and minor T allele were more recurrent in RA patients than in that of the control group. Also, TT genotype carriers had significantly lower omentin relative expression as compared to that of RA patients with other genotypes. Current results support the hypothesis that the rs2274907 might be of functional relevance due to presence of highly conserved amino acid in its vicinity [41].

More to the point, current study revealed that levels of chemerin and vaspin were found to be significantly and directly correlated to BMI. On the other hand, there existed a significant inverse correlation between omentin and apelin levels and BMI. Given the crosstalk between the adipokines’ levels and RA, these findings further verify the established link between the higher BMI and risk of developing RA presumably through modulating adipokines’ levels [88].

The study has some limitations; firstly, the sample was drawn from a population of established RA patients, who were on treatment. This might have affected the strength of correlation with disease activity markers (i.e., DAS-28, ESR, and CRP). However, markers of disease severity (i.e., RF and anti-CCP) are not affected by either disease duration or treatment [89]. Thus, the correlations with severity markers are still robust. Secondly, our sample was limited to Egyptian population in Suez Canal area. Further studies on different populations using larger sample size are warranted to clarify the precise mechanisms and role of studied adipokines and their genetic variants in RA pathogenesis.

## 5. Conclusions

The current study provides a new insight into the interaction between the investigated adipokines’ protein and expression levels and cellular mechanisms associated with RA. Indeed, current results provide evidence of elevated serum chemerin and vaspin levels, and on the contrary, decreased serum levels of both apelin and omentin in RA, and emphasizes the association of these adipokines with RA activity. To the best of our knowledge, this is the first study to investigate the association of genotypic and allelic variants in genes of chemerin rs17173608, apelin rs2235306, vaspin rs2236242, and omentin rs2274907 with RA in the Egyptian population. Current results revealed the association between chemerin rs17173608, vaspin rs2236242, and omentin rs2274907 and RA susceptibility, and on the other hand, lack of association between apelin gene polymorphism rs2235306 and RA. Accumulation of additional data from further studies will enable more comprehensive understanding of studied adipokines’ mechanism of action and permit the development of novel treatment strategies.

## Figures and Tables

**Table 1:** Demographic and clinical characteristics of RA patients.

Variables	RA Patients (*n* = 150)	Normal Range
	Mean ± SD	
Age (years)	44.29 ± 9.4	
BMI (kg/m^2^)	26 ± 2.7	
Age of onset of disease (years)	39.3 ± 10.6	-------
DAS-28	5 ± 1.1	-------
	Median (IQR)	
Duration of disease (years)	3 (2–7)	
ESR (mm/h)	47.5 (38.9–63)	(<or =20 mm/h)
CRP (mg/L)	19 (11.2–31)	(<3.0 mg/L)
RF (IU/mL)	44.6 (29.7–72.3)	(0–20 IU/mL)
RF positive cases	110	
RF negative cases	40	
Anti-CCP ab (U/mL)	45.3 (17.1–63.5)	(0–20 U/mL)
Anti-CCP ab positive cases	131	
Anti-CCP ab negative cases	19	
	Number (%)	
Treatment		
Methotrexate monotherapy	67(44.7)	
Leflonamide monotherapy	59(39.3)	
Combination (methotrexate +Leflonamide)	24(16)	

SD, standard deviation; IQR, 1st–3rd quartiles; BMI, body mass index; DAS-28, 28—joints disease activity score; ESR, erythrocyte sedimentation rate; CRP, C-reactive protein; RF, rheumatoid factor; CCP, cyclic citrullinated peptide.

**Table 2:** Primers, annealing temperature, and band lengths in genotyping of investigated SNPs.

Gene Polymorphism	Primers	Sequence (5′ to 3′)	Bands Lengths (bp)	Annealing Temp.
Chemerin rs17173608	FO	GTC AGA CCC ATG CAG TTT TCA AAC	549	54.5 °C
	RO	GAG TTC CTC TCT CAA GCA TCA GGG		
	FI (G allele)	ATT GCT ATA GTC CAG TGC CCT TCG	262	
	RI (T allele)	CCA GTT CCC TCT GTC GGC TTA A	332	
Apelin rs2235306	FO	AAG TGG TGC AGG GTA TCC TTG GGT	458	58 °C
	RO	AAG GAG CCA AGG AAG GAA CAG AGC		
	FI (T allele)	CCC CCT GCA CAC CAT CTG CTT	208	
	RI (C allele)	GGG ACA GGG ATC TAG ATG CAG GAAG	295	
Vaspin rs2236242	FO	GGA GGC AGA CCA GGC ACT AGA AA	378	55 °C
	RO	ACC ATC TCT CTG GCT TCA GGC TTC		
	FI (T allele)	AAG ACG CCG CTT CTG TGC ACT	174	
	RI (A allele)	CAC AGG GAC CCA GGA TAA CTT GCT	248	
Omentin 1 rs2274907	F	TGC CGT CCC CCT CTG GGT AGT	471	58 °C
	R	GTC AGC AGG GCA GCA AAG CAGA		

FI, forward inner; RI, reverse inner; FO, forward outer; RO, reverse outer.

**Table 3:** Disease biomarkers serum levels and adipokines protein and expression levels of control group and RA patients (*n* = 150).

Variables	Control (*n* = 150)	Patients (*n* = 150)	*p*-Value
	Mean ± SD		
NF-κB (ng/mL)	0.73 ± 0.12	3.9 ± 0.79 *	<0.0001
HIF-1α (ng/mL)	0.65 ± 0.12	4.79 ± 1.08 *	<0.0001
MMP-3 (ng/mL)	0.46 ± 0.09	3.50 ± 0.76 *	<0.0001
VEGF (pg/mL)	32.97 ± 4.88	130.1 ± 17.29 *	<0.0001
Bax (pg/mL)	44.55 ± 8.41	22.67 ± 5.23 *	<0.0001
Chemerin (pg/mL)	59.58 ± 11.24	207.80 ± 25.37 *	<0.0001
Apelin (pg/mL)	199 ± 11.42	82.78 ± 10.32 *	<0.0001
Vaspin (pg/mL)	57.11 ± 7.29	163.1 ± 11.53 *	<0.0001
Omentin (pg/mL)	258.8 ± 19.97	194.9 ± 11.63 *	<0.0001
	**Median (IQR)**		
Chemerin fold change	1	2.3 (0.7–7.4) *	<0.0001
Apelin fold change	1	0.4 (0.3–0.5) *	<0.0001
Vaspin fold change	1	6.8 (5.4–8.2) *	<0.0001
Omentin fold change	1	0.2 (0.1–0.5) *	<0.0001

SD, standard deviation; IQR, 1st–3rd quartiles; RA, rheumatoid arthritis; NF-κB, nuclear factor-kappa beta; HIF-1α, hypoxia inducible factor-1α; MMP-3, matrix metalloproteinase-3; VEGF, vascular endothelial growth factor; Bax, Bcl2 Associated X. * indicates significant difference at *p* < 0.001.

**Table 4:** Multivariate analysis of effect of RA on serum protein and gene expression levels of investigated adipokines in all study participants.

Variables	*F*	*p*-Value	Partial Eta Squared (η^2^)
Serum protein levels			
NF-κB (ng/mL)	2372.77	0.001	0.888
HIF-1α (ng/mL)	2164.41	0.001	0.879
MMP-3 (ng/mL)	2370.04	0.001	0.888
VEGF (pg/mL)	4380.32	0.001	0.936
Bax (pg/mL)	731.87	0.001	0.711
Chemerin (pg/mL)	4281.03	0.001	0.935
Apelin (pg/mL)	8555.89	0.001	0.966
Vaspin (pg/mL)	9061.17	0.001	0.968
Omentin (pg/mL)	1147.22	0.001	0.794
Gene expression levels			
Chemerin fold change	34.11	0.001	0.103
Apelin fold change	210.38	0.001	0.414
Vaspin fold change	70.38	0.001	0.191
Omentin fold change	648.40	0.001	0.685

Data were analyzed by one-way multivariate analysis of variance (MANOVA).

**Table 5:** Association between disease biomarkers in RA Patients.

	Disease Biomarkers												
Clinical Characteristics and Biomarkers	NF-κB	HIF-1α	MMP-3	VEGF	BAX	Chemerin	Apelin	Vaspin	Omentin	Chemerin Fold Change	Apelin Fold Change	Vaspin Fold Change	Omentin Fold Change
BMI	r_s_ = 0.48 *	r_s_ = 0.47 *	r_s_ = 0.46 *	r_s_ = 0.43 *	r_s_ = −0.45 *	r_s_ = 0.32 *	r_s_ = −0.46 *	r_s_ = 0.35 *	r_s_ = −0.2 *	r_s_ = 0.31 *	r_s_ = −0.26 *	r_s_ = 0.32 *	r_s_ = −0.28 *
	*p* < 0.001	*p* < 0.001	*p* < 0.001	*p* < 0.001	*p* < 0.001	*p* < 0.001	*p* < 0.001	*p* < 0.001	*p* = 0.02	*p* < 0.001	*p* = 0.001	*p* < 0.001	*p* < 0.001
ESR	r_s_ = 0.82 *	r_s_ = 0.88 *	r_s_ = 0.90 *	r_s_ = 0.88 *	r_s_ = −0.83 *	r_s_ = 0.51 *	r_s_ = −0.79 *	r_s_ = 0.58 *	r_s_ = −0.33 *	r_s_ = 0.45 *	r_s_ = −0.25 *	r_s_ = 0.72 *	r_s_ = −0.30 *
	*p* < 0.001	*p* < 0.001	*p* < 0.001	*p* < 0.001	*p* < 0.001	*p* < 0.001	*p* < 0.001	*p* < 0.001	*p* < 0.001	*p* < 0.001	*p* = 0.002	*p* < 0.001	*p* < 0.001
CRP	r_s_ = 0.81 *	r_s_ = 0.89 *	r_s_ = 0.91 *	r_s_ = 0.88 *	r_s_ = −0.78 *	r_s_ = 0.53 *	r_s_ = −0.78 *	r_s_ = 0.52 *	r_s_ = −0.31 *	r_s_ = 0.40 *	r_s_ = −0.2 *	r_s_ = 0.74 *	r_s_ = −0.28 *
	*p* < 0.001	*p* < 0.001	*p* < 0.001	*p* < 0.001	*p* < 0.001	*p* < 0.001	*p* < 0.001	*p* < 0.001	*p* < 0.001	*p* < 0.001	*p* = 0.016	*p* < 0.001	*p* = 0.001
RF	r_s_ = 0.62 *	r_s_ = 0.62 *	r_s_ = 0.62 *	r_s_ = 0.62 *	r_s_ = −0.63 *	r_s_ = 0.32 *	r_s_ = −0.58 *	r_s_ = 0.41 *	r_s_ = −0.29 *	r_s_ = 0.24	r_s_ = −0.17	r_s_ = 0.54 *	r_s_ = −0.34 *
	*p* < 0.001	*p* < 0.001	*p* < 0.001	*p* < 0.001	*p* < 0.001	*p* = 0.001	*p* < 0.001	*p* < 0.001	*p* = 0.002	*p* = 0.01	*p* = 0.07	*p* < 0.001	*p* = 0.001
Anti-CCP ab	r_s_ = 0.67 *	r_s_ = 0.71 *	r_s_ = 0.72 *	r_s_ = 0.66 *	r_s_ = −0.69 *	r_s_ = 0.38 *	r_s_ = −0.60 *	r_s_ = 0.47 *	r_s_ = −0.22 *	r_s_ = 0.39 *	r_s_ = −0.17	r_s_ = 0.56 *	r_s_ = −0.28 *
	*p* < 0.001	*p* < 0.001	*p* < 0.001	*p* < 0.001	*p* < 0.001	*p* < 0.001	*p* < 0.001	*p* < 0.001	*p* = 0.01	*p* < 0.001	*p* = 0.051	*p* < 0.001	*p* = 0.001
DAS-28	r = 0.83 *	r = 0.76 *	r = 0.75 *	r = 0.80 *	r = −0.76 *	r = 0.46 *	r = −0.76 *	r = 0.64 *	r = −0.38 *	r_s_ = 0.38 *	r_s_ = −0.27 *	r_s_ = 0.64 *	r_s_ = −0.31 *
	*p* < 0.001	*p* < 0.001	*p* < 0.001	*p* < 0.001	*p* < 0.001	*p* < 0.001	*p* < 0.001	*p* < 0.001	*p* < 0.001	*p* < 0.001	*p* = 0.001	*p* < 0.001	*p* < 0.001
NF-κB	------	------	------	------	------	r = 0.41 *	r = −0.72 *	r = 0.49 *	r = −0.37 *	r_s_ = 0.39 *	r_s_ = −0.36	r_s_ = 0.62 *	r_s_ = −0.28 *
						*p* < 0.001	*p* < 0.001	*p* < 0.001	*p* < 0.001	*p* < 0.001	*p* < 0.001	*p* < 0.001	*p* = 0.001
HIF-1α	------	------	------	------	------	r = 0.56 *	r = −0.70 *	r = 0.40 *	r = −0.30 *	r_s_ = 0.39 *	r_s_ = −0.26 *	r_s_ = 0.70 *	r_s_ = −0.32 *
						*p* < 0.001	*p* < 0.001	*p* < 0.001	*p* < 0.001	*p* < 0.001	*p* = 0.002	*p* < 0.001	*p* < 0.001
MMP-3	------	------	------	------	------	r = 0.55 *	r = −0.71 *	r = 0.43 *	r = −0.32 *	r_s_ = 0.41 *	r_s_ = −0.23 *	r_s_ = 0.72 *	r_s_ = −0.29 *
						*p* < 0.001	*p* < 0.001	*p* < 0.001	*p* < 0.001	*p* < 0.001	*p* = 0.004	*p* < 0.001	*p* < 0.001
VEGF	------	------	------	------	------	r = 0.51 *	r = −0.69 *	r = 0.49 *	r = −0.32 *	r_s_ = 0.36 *	r_s_ = −0.25 *	r_s_ = 0.70 *	r_s_ = −0.30 *
						*p* < 0.001	*p* < 0.001	*p* < 0.001	*p* < 0.001	*p* < 0.001	*p* = 0.002	*p* < 0.001	*p* < 0.001
BAX	------	------	------	------	------	r = −0.46 *	r = 0.66 *	r = −0.64 *	r = −0.31 *	r_s_ = −0.44 *	r_s_ = 0.27 *	r_s_ = −0.61 *	r_s_ = 0.32 *
						*p* < 0.001	*p* < 0.001	*p* < 0.001	*p* < 0.001	*p* < 0.001	*p* = 0.001	*p* < 0.001	*p* < 0.001
Respective adipokine protein level	------	------	------	------	------	------	------	------	------	r_s_ = 0.29 *	r_s_ = 0.37 *	r_s_ = 0.38 *	r_s_ = 0.31 *
										*p* < 0.001	*p* < 0.001	*p* < 0.001	*p* < 0.001

(r), Pearson’s correlation coefficient; (r_s_), Spearman’s rho correlation coefficient; RA, rheumatoid arthritis; DAS-28, 28-joints disease activity score; ESR, erythrocyte sedimentation rate; CRP, C-reactive protein; RF, rheumatoid factor; CCP, cyclic citrullinated peptide; NF-κB, nuclear factor-kappa beta; HIF-1α, hypoxia inducible factor-1α; MMP-3, matrix metalloproteinase-3; VEGF, vascular endothelial growth factor; Bax, Bcl2 Associated X. * significantly correlated.

**Table 6:** Distribution of genotypes and alleles of adipokine genes SNPs in RA patients and control subjects.

	Control (*n* = 150)	RA Patients (*n* = 150)	*p*-Value	OR (95%CI)
Chemerin rs17173608				
Genotypes				
TT	121	87		
TG	25	53	0.001 *	2.95 (1.70–5.11)
GG	4	10	0.040 *	3.48 (1.06–11.45)
Alleles				
T	267	227		
G	33	73	0.001 *	2.60 (1.66–4.07)
Apelin rs2235306				
Genotypes				
TT	46	37		
TC	79	85	0.282	1.34 (0.79–2.27)
CC	25	28	0.348	1.39 (0.70–2.78)
Alleles				
T	171	159		
C	129	141	0.325	1.18 (0.85–1.62)
Vaspin rs2236242				
Genotypes				
TT	75	105		
TA	61	38	0.002 *	2.25 (1.36–3.71)
AA	14	7	0.035 *	2.80 (1.08–7.27)
Alleles				
T	211	248		
A	89	52	0.001 *	2.01 (1.36–2.97)
Omentin rs2274907				
Genotypes				
AA	80	57		
AT	53	71	0.012 *	1.88 (1.15–3.08)
TT	17	22	0.103	1.82 (0.89–3.73)
Alleles				
A	213	185		
T	87	115	0.016 *	1.52 (1.08–2.14)

Comparisons were performed by chi-square test. OR = odds ratio, CI = confidence intervals. * indicates significant difference at *p* < 0.05.

**Table 7:** Adipokines genotypes and their association with disease biomarkers in RA Patients.

	Variables	DAS-28	NF-κB (ng/mL)	HIF-1α (ng/mL)	MMP-3 (pg/mL)	VEGF (pg/mL)	Bax (pg/mL)	Chemerin (pg/mL)	Apelin (pg/mL)	Vaspin (pg/mL)	Omentin (pg/mL)
**Mean ± SD**	Chemerin genotypes										
	TT (*n* = 87)	4.5 ± 0.97	3.6 ± 0.71	4.4 ± 0.87	3.2 ± 0.55	122.8 ± 15.4	24.9 ± 4.3	200.6 ± 19.3	------	------	------
	TG (*n* = 53)	5.5 ± 0.85 ^a^	4.3 ± 0.71 ^a^	5.4 ± 1.1 ^a^	4.0 ± 0.83 ^a^	139.7 ± 15.1 ^a^	20.3 ± 4.9 ^a^	217.1 ± 29.8 ^a^	------	------	------
	GG (*n* = 10)	6.4 ± 0.76 ^ab^	4.6 ± 0.53 ^a^	5.2 ± 0.69 ^a^	3.8 ± 0.54 ^a^	142.3 ± 12.1 ^a^	15.9 ± 1.8 ^ab^	221.6 ± 27.1 ^a^	------	------	------
	Apelin genotypes										
	TT (*n* = 37)	4.9 ± 1.2	3.8 ± 0.88	4.7 ± 1	3.4 ± 0.81	127.7 ± 19.8	23 ± 5.5	------	84.1 ± 9.2	------	------
	TC (*n* = 85)	5.1 ± 0.99	4 ± 0.70	4.9 ± 1.1	3.5 ± 0.71	131.9 ± 15.2	22.4 ± 5.2	------	82.1 ± 10.6	------	------
	CC (*n* = 28)	4.8 ± 1.3	3.9 ± 0.92	4.7 ± 1.2	3.5 ± 0.84	127.8 ± 19.5	23 ± 5.2	------	83 ± 11	------	------
	Vaspin genotypes										
	TT (*n* = 105)	5.1 ± 1.1	4 ± 0.80	4.9 ± 1.1	3.6 ± 0.79	131.8 ± 17	22.1 ± 5.3	------	------	164.8 ± 11.9	------
	TA (*n* = 38)	5 ± 1	3.9 ± 0.73	4.7 ± 1	3.4 ± 0.67	128.4 ± 17.6	23.1 ± 4.9	------	------	161.3 ± 9.1	------
	AA (*n* = 7)	3.5 ± 0.63 ^ab^	3 ± 0.55 ^ab^	4 ± 0.51	2.8 ± 0.30 ^a^	113.5 ± 11.38 ^a^	28.5 ± 0.76 ^ab^	------	------	148.4 ± 4.7 ^ab^	------
	Omentin genotypes										
	AA (*n* = 57)	4.1 ± 0.88	3.3 ± 0.63	4.1 ± 0.69	3 ± 0.36	118.2 ± 13.7	25.7 ± 4	------	------	------	199.7 ± 9.6
	AT (*n* = 71)	5.4 ± 0.79 ^a^	4.2 ± 0.66 ^a^	5.1 ± 1 ^a^	3.8 ± 0.75 ^a^	135.5 ± 15.2 ^a^	21.4 ± 4.9 ^a^	------	------	------	193.6 ± 11.2 ^a^
	TT (*n* = 22)	6 ± 0.85 ^ab^	4.5 ± 0.59 ^a^	5.6 ± 1 ^a^	4.1 ± 0.72 ^a^	143.4 ± 13.8 ^a^	19 ± 5 ^a^	------	------	------	186.4 ± 12.2 ^ab^
**(IQR)**	Variables	ESR (mm/h)		CRP (mg/L)	RF (IU/mL)	Anti-CCP ab (U/mL)	Chemerin fold change		Apelin fold change	Vaspin fold change	Omentin fold change
	Chemerin genotypes										
	TT (*n* = 87)	41 (30–49.5)		13.6 (7.9–23)	38.9 (19.5–50.8)	34.5 (13.5–52.6)	1.1 (0.64–6.2)		------	------	------
	TG (*n* = 53)	61 (47.8–80) ^a^		30.1 (17.3–43.1) ^a^	61.7 (42–93) ^a^	48.2 (23.3–73.2) ^a^	3.1 (0.64–7.5)		------	------	------
	GG (*n* = 10)	101.8 (51.5–118) ^a^		52.5 (19–65.5) ^a^	190 (36.4–242) ^a^	140.4 (36–204.5) ^a^	6.5 (4.1–18.8) ^a^		------	------	------
	Apelin genotypes										
	TT (*n* = 37)	45 (34–62.5)		17.2 (7.7–29.9)	42.5 (34–71)	34 (13.8–62.6)	------		0.32 (0.20–0.42)	------	------
**Median (IQR)**	TC (*n* = 85)	48.5 (40–62.5)		21.7 (12.7–30.6)	45.2 (28.8–72.3)	45.6 (18.4–63.4)	------		0.32 (0.27–0.38)	------	------
	CC (*n* = 28)	47.8 (30.1–67.9)		20.1 (8–39.2)	40 (18–100)	46.3 (14.6–90.2)	------		0.35 (0.24–0.38)	------	------
	Vaspin genotypes										
	TT (*n* = 105)	48.5 (39–68.5)		22.7 (11.4–35.4)	44.4 (28.8–69.5)	45.7 (20.2–65.4)	------		------	6.9 (5.3–8.5)	------
	TA (*n* = 38)	46.5 (39.9–60)		18.2 (11.3–27.5)	45 (33–89.8)	42 (16.9–61.7)	------		------	6.8 (5.8–7.7)	------
	AA (*n* = 7)	16 (15–41.7) ^ab^		10 (4.9–19)	53.5 (28–79)	13.7 (13–24.3) ^a^	------		------	4.7 (2–6.3) ^a^	------
	Omentin genotypes										
	AA (*n* = 57)	39 (15.5–43.5)		10.6 (5.2–17.2)	34.8 (18–52.4)	17.1 (13.5–45.7)	------		------	------	0.36 (0.13–0.55)
	AT (*n* = 71)	56 (43.9–68.5) ^a^		23.6 (15.1–36.2) ^a^	43.4 (33.9–72.8)	45.7 (24.3–65.2) ^a^	------		------	------	0.17 (0.10–0.45)
	TT (*n* = 22)	78 (48.5–108.6) ^a^		37 (21.6–51) ^a^	62 (46–190) ^a^	65.4 (47.1–140.4) ^a^	------		------	------	0.10 (0.06–0.22) ^a^

Data are presented as mean ± SD or median (IQR). Comparisons were performed by one way ANOVA and Tukey post-hoc test for parametric data. Kruskal-Wallis test and Dunn’s multiple comparisons test were performed for non-parametric data. RA, rheumatoid arthritis; SD, standard deviation; IQR, 1st–3rd quartiles; DAS-28, 28—joints disease activity score; ESR, erythrocyte sedimentation rate; CRP, C-reactive protein; RF, rheumatoid factor; CCP, cyclic citrullinated peptide; NF-κB, nuclear factor-kappa beta; HIF-1α, hypoxia inducible factor-1α; MMP-3, matrix metalloproteinase-3; VEGF, vascular endothelial growth factor; Bax, Bcl2 Associated X. ^a^ significantly different from corresponding homozygots of the major allele, ^b^ significantly different from corresponding heterzygotes at *p* < 0.05.

## Data Availability

The datasets used and analyzed are available from the corresponding author on reasonable request.
